# Governing therapy choices: Power/Knowledge in the treatment of progressive renal failure

**DOI:** 10.1186/1747-5341-1-12

**Published:** 2006-12-04

**Authors:** Dave Holmes, Amélie M Perron, Marc Savoie

**Affiliations:** 1 School of Nursing, Faculty of Health Sciences, University of Ottawa, Ottawa, Canada; 2 Renal Division, Baxter Corporation, Montréal, Canada

## Abstract

This article outlines the struggle between the power of the health care professional and the rights of the individual to choose freely a modality of treatment. Nurses are instrumental in assisting patients in making the best decision for a therapy they will have to assume for the rest of their lives. In guiding patients' decision, nurses must take into account these unavoidable contingencies: changes in lifestyle, nutritional restrictions, level of acceptance, compliance issues, ease of training and availability of support/facilities. Ensuring that the patient makes an informed decision is therefore an ongoing challenge for nurses as they are taking part in a delicate balancing act between not directly influencing the patient's decision while making sure the patient is accurately informed.

## Background

"Is it not the supreme exercise of power to get another or others to have the desires you want them to have – that is to secure their compliance by controlling their thought and desire" [1].

Kidney disease information can be found on several Internet sites. Some of this information is adequate, but most of it is questionable [2]. Consequently, when a patient is faced with making the choice of a therapy to alleviate the effects of a chronic disease, he or she usually turns to health care professionals for reliable advice [3]. Once a patient has reached the End Stage Renal Disease (ESRD), only three options are available: organ transplant, dialysis, or no treatment and unavoidable death.

Patients must be given detailed information to help them select the particular mode of replacement therapy that will maximize their quality of life and keep them as active as possible [4-6]. The concepts of Integrated Care (promoting peritoneal dialysis as an initial therapy) [7-9] and pre-dialysis planning (to slow down the progression of the disease and empower the patients in making informed decisions) are the current trend [10-13]. Yet, many patients still present themselves at emergency clinics in a state of advanced renal insufficiency requiring immediate attention, or are in crisis upon learning that they have been stricken with a chronic illness and they now have to choose a replacement therapy option.

In the emergency settings, a majority of patients will commence haemodialysis (HD) to correct their state of imbalance. While undergoing HD, these individuals not only need to come to grips with the fact they have a chronic illness, but must also decide if they wish to pursue this treatment or choose an alternative mode of therapy [14]. The majority opts to stay on haemodialysis even though other therapies available may be more suitable to their lifestyles. Significantly, some patients said they had no involvement in the decision: "Patients stated that they were too sick to make a decision, and that the decision was made for them..." [3].

Employing the example of the currently available therapies for Chronic Kidney Disease (CKD), this article will outline how different modes of discipline and care may provide strategies for governing individual bodies. We will concentrate on the extent to which the boundaries of disciplinary and pastoral power are often blurred for health care professionals, and while acknowledging that this analysis would be applicable to other health care professionals who are involved in the decision-making process of patients facing treatment options, we will focus our analysis on nurses, because they have close and ongoing contact with these patients. Using the work of late French philosopher Michel Foucault, we will explore the theoretical aspects of bio-power and governmentality within the scope of the nurse-patient relationship to illustrate nurses' influence in shaping patients' decisions.

## Governing the Self: Bio-power and the Management of the Disabled Body

Traditionally power is what is seen, what manifests itself and paradoxically 'finds its strength in the movement through which it is deployed' [15]. In this regard, Foucault's works on disciplinary and pastoral powers are evocative. Foucault argues however that since the 17^th ^century we are witnessing a whole new approach in the management of individuals and populations. For over three centuries now "western man was gradually learning what is it meant to be a living species in a living world, to have a body, conditions of existence, probabilities of life, an individual and collective welfare, forces that could be modified, and a space in which they could be distributed in an optimal manner" [16].

This form of power is two-fold: anatomo-politics of the human body (training, power intensification and distribution, energy management) and bio-politics of populations (demographics, estimation of resources to inhabitant's ratio, wealth appraisal, birth rate, longevity, health, etc.) [17].

Anatomo-politics is the first pole that has developed during the 17^th ^century and it targets the body as a machine that can be trained and disciplined. Anatomo-politics refer to the disciplinary dimension of bio-power. Through its own techniques discipline shapes many different types of individualities (cellular, organic, genetic, etc.). Discipline is a special technique that targets individuals both as objects and instruments necessary for its practice [15].

Bio-politics of populations emerges at the end of the 18^th ^century. It makes up the second pole of bio-power and refers to the regulations and control to which a collective body of individuals ("objectified" and "subjectified" through varying techniques) is subordinate. At this point, discipline (anatomo-politics) is not eliminated from the State's priorities. Quite the contrary, "the managing of a population not only concerns the collective mass of phenomena, the level of its aggregate effects, it also implies the management of population in its depths and its details [...] the notion of government of population render all more acute equally the necessity for the development of discipline" [18].

Articulation of both dimensions of bio-power allows for the proliferation of political technologies, which invest the body, health, eating habits, lodging standards, life conditions, the entire space of existence [19]. Far from occupying a strictly repressive function, bio-power can 'optimize, administer, and multiply life, subjecting it to precise controls and comprehensive regulations' [16]. This new form of power describes, measures, estimates and institutes hierarchies; it defines norms in order to detect differences: normalizing society as we know it today represents a historical effect of a technology of power over life [19].

Throughout history, nurses have been involved in the governance of individual bodies through a vast array of power techniques producing effects that construct subjectivities, such as establishing standards for the 'good patient', the 'healthy citizen', and the 'caring mother', among others [20]. In an era of Bio-power, the individual body is the focus of analysis and thus is constructed through the powerful discourses (including practices) of health care professionals. Many power techniques are involved in the construction of docile, obedient and compliant bodies. Disabled bodies constitute a target for professional gaze and scrutiny. Whatever the 'impairment', whether physical or psychological, professional interventions are likely to target the individual.

The uncovered body is most likely to fall under the unrelenting gaze and intervention of professionals [21]. For example, individuals suffering from progressive renal failure come in direct contact with nurses working in the field of nephrology. The disabled body of the patient comes in contact with an agent, the nurse, and might find itself objectified and subjected to techniques of power: disciplinary and pastoral, while the patient chooses a therapeutic regime that fits him/her well.

## Disciplinary power

Disciplinary power is one form of power among others (sovereign, pastoral), exercised over an individual or many persons in order to produce effects on their conduct, habits, and attitudes and ultimately help them achieve particular skills and new ways of thinking or to make them ready for instruction [22]. Disciplinary power is subtle and does not need violence in order to be effective. Some authors argue that modern bodies are not physically constrained; rather they have legal rights, and as such direct forms of control are not easily acceptable [23]. In fact, disciplinary power not only produces docile bodies, but new subjectivities as well. It extracts forces, enhances them, and makes optimal use of their capacities. Disciplinary power operates through an impressive set of tools such as hierarchical observation (unrelenting surveillance of 'captive' clients, clients at risk, and communities); normalizing judgement (creation of norms, (micro) penalties, and rewards); and examination (clinical gaze, use of time and space, creation of individual cases) [24].

Self-regulation is a dominant form of social control and nurses' therapeutic practice is currently based on the principles of self-care, which foster patient self-regulation [25]. Through peritoneal dialysis as an alternative treatment regime to hospital dialysis, individuals could reach self-regulation by being involved in a therapeutic enterprise in which nurses would promote self-surveillance and self-awareness through pastoral care. Along with disciplinary power, pastoral power plays a major role in the governance of individuals and populations.

## Pastoral power

Pastoral power has developed in Christian societies around the 3rd century AD, and has become an important form of power. It requires a person to serve as a guide for another. Through this benevolent power, 'the guide' cares for others: 'The pastoral model is adopted and vastly elaborated by Christianity, as the care of souls' [26]. Introduced in the Western world along with Christianity, pastoral power is an individualising form of power [19]. It lies in a power technique that must penetrate souls, decode hearts, and reveal the most intimate secrets. It seeks disclosure of consciousness; it penetrates the soul and acts upon it, to ultimately direct it [19].

Pastoral power is another form of power regularly used by nursing staff which is part of a control mechanism that produces a *savoir *on the governed subject. This acquired savoir, through its main tool, the confession, is codified and integrated within, but not exclusive to, the specialised discourses of medicine, psychiatry, psychology, sexology, criminology, and nursing care. The strange secrets of the individual, exposed to professional scrutiny, are incorporated in expert professional discourses [27]. This is often the starting point for labelling the client as normal or deviant. Professional intervention is likely to take place in these circumstances [28].

If detailed knowledge over individuals is required for this form of power to be effective, the 'therapist' will rely on various techniques to uncover these secrets [19]. At the same time, clients must open themselves up to the other and are in part subjugated to avowal. This trustful, therefore unconditioned, obedience, as well as unrelenting examination and confession, form a powerful combination. The knowledge of clients, hidden until now in their souls, constitutes an important element in the governance of others [19].

Confession, an essential element of pastoral power, provokes an intensification of regulatory controls over citizens. Moreover, "professions investigating psychic states could be extremely useful to a bio-power construct intent on managing the variables associated with population" [29]. Power always questions, inquires, records, and institutionalises truth. Furthermore, it does so in a professional way [27]. In fact, the pastoral use of confession, introspection, and self-examination is found today not only in churches or sects, but in the day-to-day work of health care professionals, including nurses. These techniques are part of the therapeutic tools used for counselling, personality modification, personal development, health education and, of course, psychiatric care. "This exercise of self-government serves as an instrument of the government of their conduct" [27]. In short, pastoral power is a form of power "which makes individuals subject, subject of someone else by control and dependence, and tied to his own identity by a conscience of self-knowledge" [29]. 'There is no need for arms, physical violence, material constraints. "Just a gaze, an inspecting gaze, a gaze... which each individual thus exercises this surveillance over and against himself" [27].

The productive effect of pastoral power is often obtained despite the objectives formulated by the health professional. Sometimes, patients come to believe that their thinking arises from their own concerns and not from others' (the therapist for instance). They are becoming aware of certain phenomena that they are experiencing. But patients become aware through the lens and evaluation of someone else, most often the therapist. "Is it not the supreme exercise of power to get another or others to have the desires you want them to have, that is, to secure their compliance by controlling their thought and desire"' [1]. In psychiatric care, for instance, nurses are actively involved in this form of power through therapeutic communication in individual therapy.

Foucault's work on bio-power and governmentality show that the centralization of power within the State exists but could not be limited to this juridico-discursive view. Power involves surveillance and meticulous examinations through which human beings are turned into objects and subjects with specific objectifying and subjectifying techniques. Government understood as the government of other's behaviours substitutes for the State. Through the concept of governmentality, Foucault sets a new space for reflecting on human behaviour. It includes the domains of ethics, government, and politics, of the government of self, others, and the state, of practices of government and practices of the self, of self-formation and political subjectification, that weaves them together without a reduction of one to the other[30]. Government implies an active process that binds political rationales (government's aims) with techniques of behaviour government (practices and techniques for the transformation of actions, conditions and subjects within a specific field of intervention) [31]. The articulation of government rationales and behaviour government techniques lies for the most part on a body of knowledge specific to a field of intervention. This implies the involvement of a professional expert which acts as a mediator between political goals and autonomous activities of an individual onto whom the professional intervention is carried out. As such, power framed within governmentality goes beyond the State and its apparatus. It relies on other agents which (unconsciously perhaps) ensure optimal functioning of this new form of government [24]. This allows the State to govern its subjects from a distance [31].

## Nurses as agents of governmentality

Hospitals and outpatient clinics constitute typical settings where government health imperatives and clients' lifestyles collide on a daily basis. To be fully understood by their target audience, these government imperatives must often be translated into lay language and, given their professional education and clinical expertise, health care professionals in general and nurses in particular are in an excellent position to fill the role of translator thus helping clients to make sense of the condition from which they suffer and giving relevant meaning to an illness, especially when it is chronic in nature, (e.g., kidney disease). These professionals are also able to educate clients about treatment options and what these mean in terms of lifestyle changes. From a Foucauldian perspective, one could consider nurses as agents of governmentality who relay health guidelines that carry benefits on a larger scale (collective health, resource management, economic performance, etc.) [17].

Because of their role in the health care system, and because of the authority invested in them, nurses learn a great deal about their patients. As a direct result of the ongoing evaluation and observation that nurses perform on a continuous basis, a considerable amount of knowledge which helps them "objectify" patients is collected – patients are described in terms of physiological systems, abilities and limitations (physical or other), resources, needs, desires, etc. – knowledge that helps them to identify new sites of intervention, in other words, new sites where power may be exercised [18]. For example, nurses must ensure that patients fully understand how to care for themselves at home and that they will comply with treatment orders, if these patients are to be discharged safely following a diagnosis of kidney failure. As a consequence, clients may need further education about the etiology of their condition, the symptoms to watch for, complications that will require emergency care, dressing changes or wound care, medication management, and necessary lifestyle changes, especially regarding nutrition and physical activity. Nurses are responsible for informing clients about all aspects of their condition including the resulting treatment. Clients usually comply with these directives because of the authoritative status which has been invested in these nurses.

From Foucault's perspective, clients are thus, simultaneously, made the objects and subjects of power during the provision of health care. They are objects because nurses (among others) exercise power and authority to accumulate knowledge about them and they are subjects because, through activities such as education and therapy, they must acquire the tools and skills to take charge of their condition and to manage their own care with minimal input from designated health care providers.

This health-care provision process aims for the fullest recovery possible. Based on the available guidelines, health care providers have a clear idea of "where clients should be and what they should do" in terms of self-management following a particular diagnosis. However, while acting in what is perceived as the best interests of clients, care providers may not be including patient autonomy regarding desires or preferences as part of the equation, especially when these are not consistent with current health guidelines. The following section will examine the implications of this oversight in the care process.

## The Ethics of Self-Determination

While most health care workers yearn to practice their profession in an ethical manner, the works of Foucault and other critical writers are useful in questioning daily practices as ethical ones. In nursing, respect for patient's autonomy (self-determination) is ever present in codes of ethics. It is the nurses' responsibility to ensure that patients have all the necessary information to make informed decisions. As delineated above, this is especially true when such decision will impact the rest of a patient's life, such as is the case in the context of kidney disease. However, through the lens of bio-power, one wonders how one can be ethical and truly endorse a patient's right to self-determination. The way in which a nurse presents a patient with the information needed to select a modality of treatment will impact this patient's decision. The patient will thus become "subjectified" to the nurse's expertise and authority, even when the nurse is unaware of exercising such authority.

In the field of health care, more and more emphasis is being placed on a practice known as self-care, that is, on clients' participation in their own care. This practice begins at home, when the individual chooses to lead a life that will protect him/her from illness. This refers to Foucault's analysis of anatomo-politics, where one imposes restrictions on one's life for the greater (collective) good [18]. As such, one endorses the role of an active and responsible citizen who will not be a burden on the health care system. While it seems individuals exercise their right to self-determination, repetitive images and discourses are presented to them on a daily basis to ensure one choice over another (e.g. fat-free food choices, tobacco-free lifestyle, safe sex, breastfeeding, etc.) [17]. This is done by ensuring that other benefits, sometimes aesthetic in nature (e.g. the need for a slimmer body), are juxtaposed to the main one (i.e. healthy life, being a "good" parent). In order to make possible the incorporation of guidelines and policies into daily routine, it is thus important to convince each individual that what they are attempting to attain is in fact in their own interest [32]. Idealizing opinions and behaviours facilitates the management of oneself and promotes the construction of the "moral", conscientious citizen. Anatomo-politics and bio-politics combine the discipline of the self with the promulgation of desirable behaviours and therefore allow for the making of individuals as moral and ethical beings as their behaviours serve both the individual and collective good [17].

It may be argued that this need to embody morality is also true when one seeks care for a chronic disease such as kidney failure. Patients who are facing difficult decisions with regard to long-term therapy are presented with several modalities that will impact their lives differently. As explained above, the pros and cons of these choices are often presented to patients in a manner that suggests the desirability of one option over another, especially when such option is deemed less costly by the institution that provides it. This particular option however may not suit a patient's desires, lifestyle or values which could lead to reduced adherence to treatment and diminished quality of life. A patient may *sense *however that he or she should consider it above the other options and may *feel *the need to reconcile his or her preferences with the care team's inclination. While it is argued that disciplinary power does not need violence in order to be effective [23], other authors contend that on the contrary it conceals new forms of violence that have never been equaled [17,20,33] because it comes from within. This new form of violence is thus subtle and – more importantly – acceptable. In this sense, the boundary between one's desires and another entity's directives is blurred to the point that the latter becomes (part of) the former. Health care professionals in general and nurses in particular must thus be aware of the effect they may have on their patient's decisions in order to truly integrate a patient's right to self-determination in their practice.

## Final Remarks

The clinical nursing gaze assumes considerable social power in defining reality, guiding patients' choices and in identifying deviance and social disorder [20]. The notion of compliance implies that if the disabled body of the patient is portrayed as passive [20], we must keep in mind that the same disabled body can resist professional power in numerous ways.

Reviewing the practice of nursing and the provision of health care through the lens of Bio-power and governmentality could be perceived by many as a threatening experience. We previously have commented that the use of Foucauldian concepts 'can generate a form of critical immobilism' [see for instance [20,25]] because governmentality links together repressive and constructive ways of exercising power. The deterministic nature of 'power everywhere' and the sense of being governed even through our freedom generate strong and emotional responses – a need to escape – especially because moral attributes traditionally have been attached to different ways of governing. We are used to searching for 'the right way' to care for patients and for being ethical in our professional relationships, especially those governing patients' choices. For instance, compliance through disciplinary power becomes an imperative for patients suffering from life-threatening diseases which can be managed by medication. However, the concept of governmentality challenges at least two assumptions that are taken for granted in nursing: patient empowerment becomes a call for self-regulation; and ethics becomes politics, in the sense that despite one's endorsement of ethical principles, one cannot escape the complex power relations that permeate and regulate all social interactions.

The concept of governmentality should be seen as a valuable tool for deconstructing nursing as an apolitical practice and a powerless profession. However, it should also help us to envision alternative ways of practising nursing. This 'reconstruction' should be permeated by critiques that emerge from governmentality because this concept reminds us that competing discourses are constantly reshaping nursing and health in the social, economic, and political arenas. In order to propose new and critical utopias, we suggest combining governmentality with critical social theories. To understand power without being able to identify possible transformations derived from this Foucauldian perspective will not lead nursing into critical 'clinico-political' action.

## Competing interests

The author(s) declare that they have no competing interests.

## Authors' contributions

DH, AP & MS have all participated in the design and the writing of the manuscript, as well as the editing of the final draft. All authors read and approved the final manuscript.
